# Toxic Effect of Antipyrine

**Published:** 1889-06

**Authors:** 


### EDITORIAL.

### TOXIC EFFECT OF ANTIPYRINE.

Dr. Tuckzek reports a case [*in Berliner klinische Wochenschrift*] in which toxic symptoms occurred after the use of antipyrine. A boy of four years with whooping-cough was treated with antipyrine; 0.4 gram at a dose was given three times a day, making 1.2 grams a day. The drug was taken for three weeks with benefit to the cough and without any apparent bad effects. All at once he became sleepless, suffered from jactation and vomited, at first after coughing and then spontaneously. He became sleepy, apathetic, and about eighteen hours after the first unpleasant symptoms he had an epileptic seizure. This was followed by others in rapid succession and for the next thirty-six hours he had about thirty seizures. At this time there was a "peculiar type of breathing, snuffling inspirations with long pauses after the style of the Cheyne-Stokes phenomena." The pulse was slow, of high tension and not quite regular. Temperature in the rectum was subnormal, 36°7.C. [98° Fah.] The pupils were dilated and reacted to light. The urine, passed in bed, smelled strongly of acetone. Towards evening of the first day a faint eruption resembling scarlatina appeared on the abdomen, cheeks and ears, but this soon disappeared. The urine was acid, with specific gravity 1028, but contained neither albumen nor sugar. It smelled strongly of acetone, and when tested with nitro-prusside of sodium it gave a very intense reaction.*

Investigation of the chest and abdomen gave negative results.

There seems to be no reason for suspecting disease of the brain or of any other organ and the conclusion seems irresistible that the trouble was due to the effect of the antipyrine.

The presence of the acetone is an interesting feature of the case. Its source and its effect on the symptoms is not clear.

Dr. Tuckzek has devoted some time to looking over the literature of the subject, and has summarized some of the most interesting of the unpleasant symptoms after taking the drug.

A remarkable peculiarity of the drug is that, as in the present case, it may be well borne for a time, when suddenly unpleasant symptoms show themselves. Sudden collapse is among these unpleasant symptoms. Several cases of sudden death after taking the drug are mentioned, probably due to its action.

Dangerous symptoms are especially frequent in children.

Symptoms of an eruptive nature occur in about ten per centum of the cases.

Nausea, or nausea and vomiting, are not at all uncommon, even after very small doses.

Symptoms due to disturbance of the nervous system stand out prominently. The most common is a feeling of relaxation or weakness. This may pass on to somnolence or apathy, with involuntary passage of the urine. Conditions of excitement may occur, or symptoms of intoxication; anxiety, a frightened feeling, delirium, chilliness, cyanosis, arythmia of the heart's action, lack of breath, subnormal temperature, dizziness, headache, ringing in the ears, dilatation of the pupils, and transitory amaurosis make up a long list of more or less alarming symptoms which are common.

It would not be difficult to add to this list of symptoms given by Dr. Tuckzek,

---

*This, not very common, test for acetone is made by adding nitro-prusside of sodium and ammonia, or carbonate of ammonia to the suspected fluid. If acetone is present the fluid will gradually become first rose-red, then a more or less dark violet wine-red. The reaction takes place more quickly by shaking in the air or by the addition of a drop of strong acid, but the fluid must still remain alkaline. By heating, the color disappears to return on cooling.

but we will mention only one case which came under the observation of the writer.

A well-known physician in this city, while in his usual good health took five grains of antipyrine as an experiment. Within three minutes, he says, he began to sneeze violently, and sneezed fifty or sixty times within a minute or two. By that time the nasal passages were occluded by the swelling of the mucous membrane and he could sneeze no more. He then began to suffer from dyspnœa, and for a minute or two he was greatly alarmed for fear of suffocation. After a while this passed off, and he had leisure to examine himself. He found the nasal mucous membrane so much swollen that no air could pass through the nose, the posterior wall of the pharynx was fiery red and swollen so as to project beyond the velum of the palate. Five or six hours after taking the drug he called at the office of the writer and related the circumstances. The worst of the symptoms had disappeared, but the nasal passages were obstructed, and the mucous membrane was still quite red. The posterior wall of the pharynx had a spongy look, the swelling was bounded by a sharp line just above the upper border of the tongue. This all disappeared within a few days.

These cases show that antipyrine is unsafe, even when watched, and that the practice by the laity of using it without advice, which appears to be growing, should be severely condemned.

